# Primary myxoid chondrosarcoma of the breast

**DOI:** 10.1308/003588414X13824511649210

**Published:** 2014-01

**Authors:** A Farahat, N Magdy, A Elaffandi

**Affiliations:** National Cancer Institute, Cairo,Egypt

**Keywords:** Mesenchymal tumours of the breast, Primary chondrosarcoma, Breast conservative surgery, Breast cancer

## Abstract

Primary breast chondrosarcoma has been rarely reported in the literature. Conservative breast surgery has never been part of the management of previously reported cases. Surgery remains the mainstay management of such a disease as it is resistant to chemotherapy and radiotherapy. In this report, we present a case of rare primary myxoid chondrosarcoma of the breast that was managed successfully with a conservative approach.

Non-epithelial breast malignancies are rare, accounting for less than 1% of all breast tumours.^1,2^ Breast sarcomas have hardly been reported in the literature (less than 0.1% of all malignant breast tumours).^1,[Bibr CIT2]2^ Primary chondrosarcomas of the breast are the most rare among this group.^2^

There is no standard clinical presentation. However, all the described cases have been reported in patients over 50 years of age, with a rapid rate of growth and reaching a relatively large size. In spite of locally aggressive behaviour, none of the reported cases showed any signs of skin infiltration or nodal involvement.

There is still only limited knowledge on the best surgical approach in the management of such cases. The role of neoadjuvant or adjuvant therapy has yet to be defined.^1–3^

## Case history

**Figure 1:**
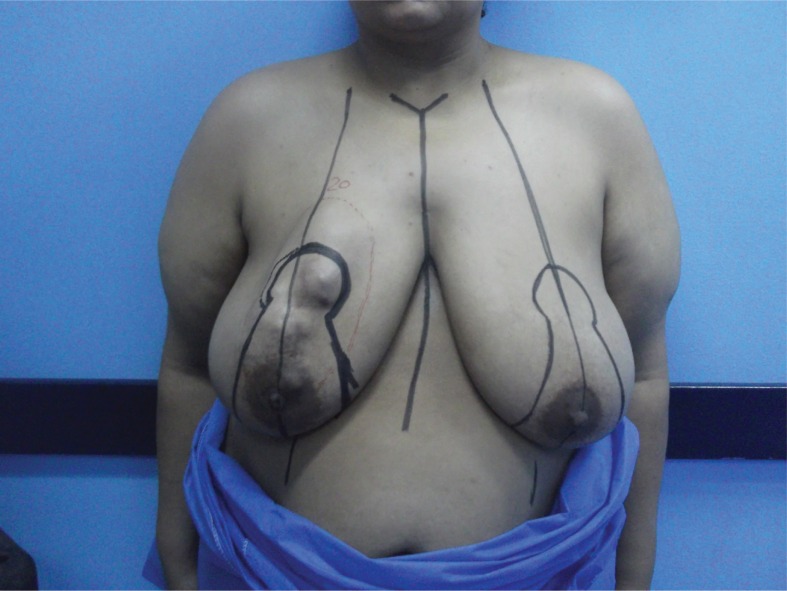
Preoperative photograph showing right breast mass and incision lines for planned conservative breast approach

A 35-year-old woman presented to the surgical outpatient clinic of the National Cancer Institute at Cairo University with a rapidly growing right breast mass (Fig 1). She had no significant past medical history and no family history for cancer. Clinical examination showed a palpable, relatively mobile, painless breast mass situated between the upper quadrants with no overlying skin involvement. The mass was hard in consistency, nearly measuring 10cm × 10cm with a well defined irregular outline. Examination of the axilla and contralateral breast was unremarkable. Ultrasonography showed a large well defined heterogeneous hypoechoic lobulated mass measuring about 10cm × 8.8cm in diameter with no ipsilateral axillary lymphadenopathy. Imaging of the contralateral breast and axilla was insignificant. Triple assessment was completed by performing a core tissue biopsy that showed a myoepithelial lesion. Complete excision was therefore recommended in our multidisciplinary team (MDT) meeting.

**Figure 2:**
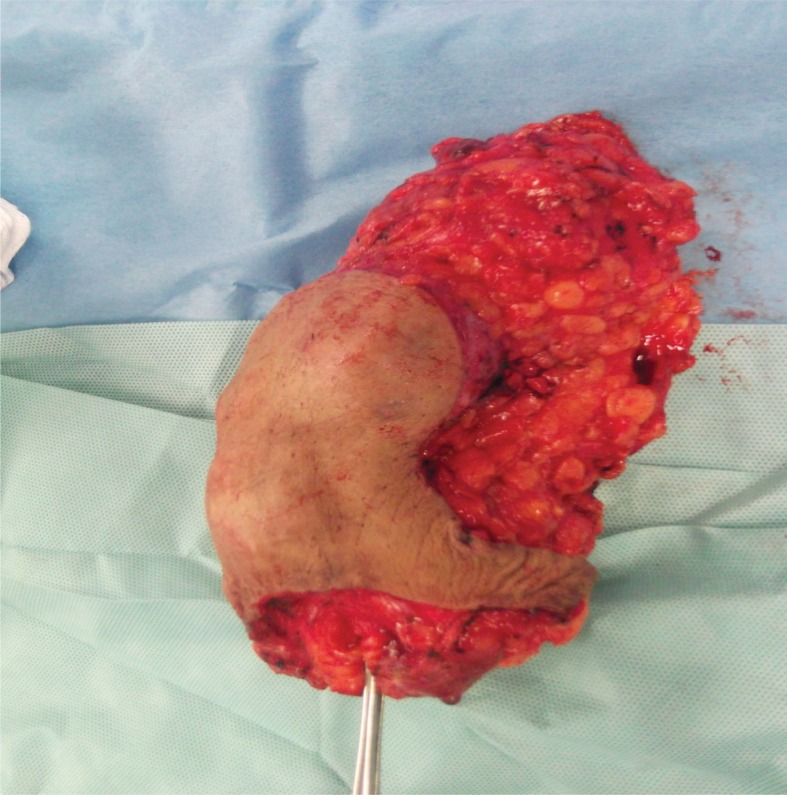
Gross specimen following excision

**Figure 3:**
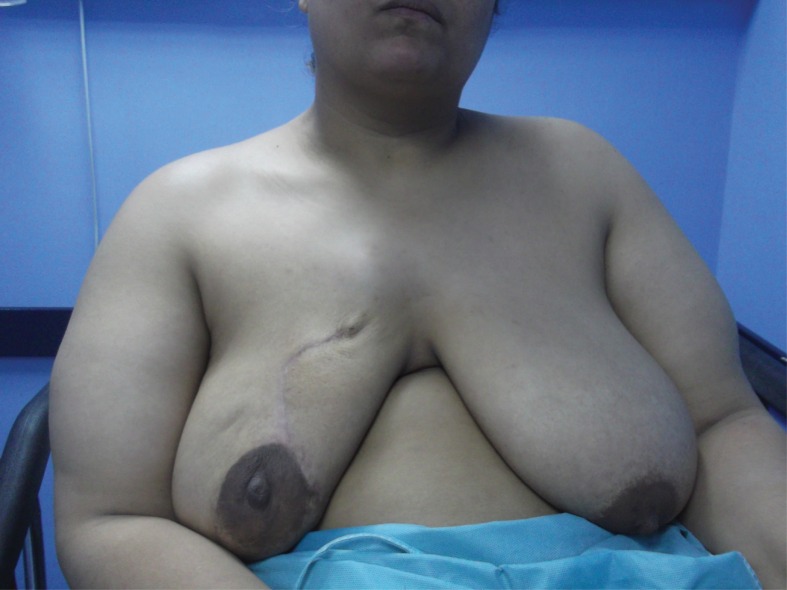
Follow-up photograph one month after conservative breast surgery. There was no need for contralateral reduction mammoplasty. (No major discrepancy in breast symmetry.)

Our provisional diagnosis following discussion in the MDT meeting was phyllodes tumour of the breast, and a plan for complete excision and reconstruction was established. Incision was planned preoperatively with an inferior-based breast reduction plan (Fig 1). Complete excision of the mass was carried out with negative safety margins (Fig 2) confirmed by intraoperative frozen section. Local glandular flaps were used to reconstruct the remaining breast volume (Fig 3) without major discrepancy in breast symmetry. The postoperative course was uneventful.

**Figure 4:**
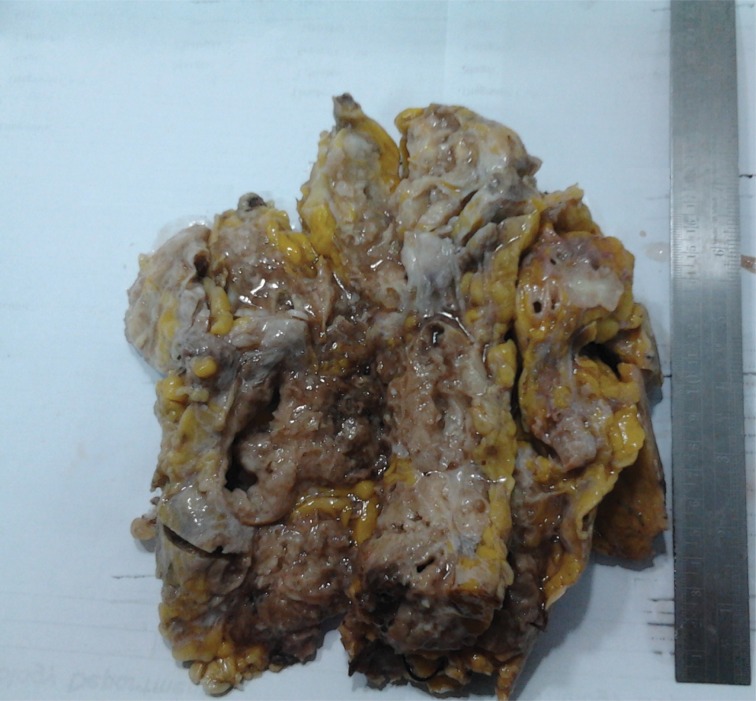
Gross specimen: tumour measured 19cm × 9cm × 9cm; lobulated, firm, friable, whitish mass with few chondroid and wide myxoid areas with focal cystic change and haemorrhagic foci

**Figure 5:**
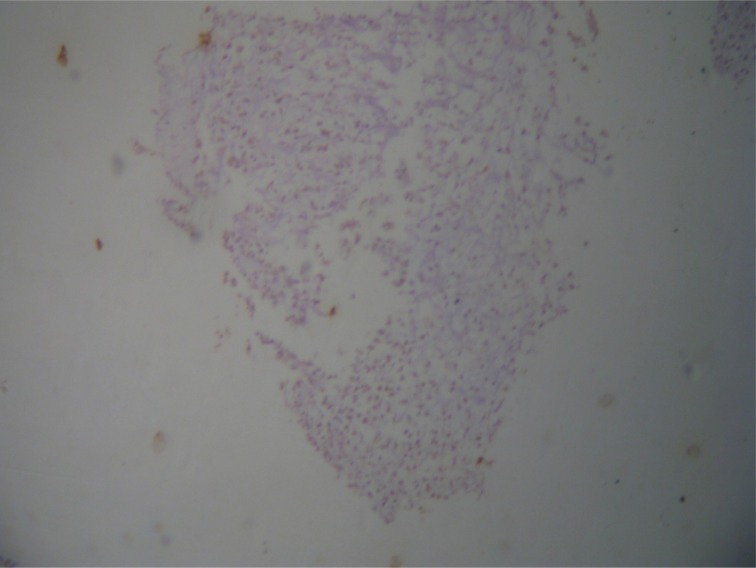
Microscopic picture showing nodules separated by fibrous septa and populated by cellular groups of malignant small to medium-sized round cells lying in lacunar spaces and having vesicular nuclei and prominent nucleoli, disposed in a loose myxoid, focally chondrohyaline matrix. (Few tumour nodules showed peripheral rim of calcific shell. No epithelial component could be detected and the possibility of a malignant phyllodes tumour, a carcinosarcoma or a metaplastic carcinoma was therefore excluded.)

**Figure 6:**
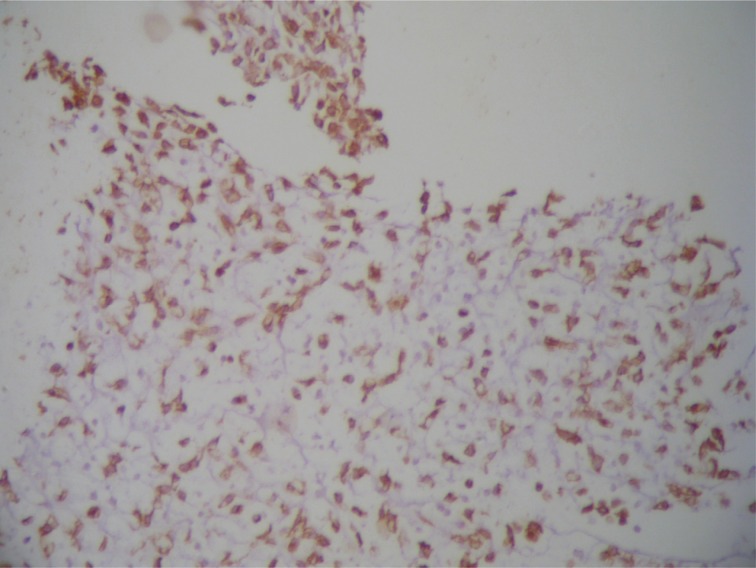
Immunohistochemistry showing a positive reaction of the tumour cells to S100 protein, and a negative reaction to casein kinase, calponin and actin

Gross and microscopic pictures of the excised mass (Figs 4–6) were consistent with the picture of rare extraskeletal chondrosarcoma, myxoid type. The patient has not received any adjuvant therapy. She is currently under close follow-up and has remained disease free for the last 15 months.

## Discussion

Primary breast sarcoma is a rare entity accounting for less than 1% of all malignant breast tumours.^1,2^ Up until 2011, only seven cases of chondrosarcoma of the breast had been reported in the literature.^1^ All reported cases presented similarly with a rapid rate of growth, reaching a relatively large mass in the breast within a short period of time. Such a rapid onset has never been reported as being associated with skin involvement, regional lymphadenopathy or even any systemic disease.^4^

It is obvious that all of the reported cases had an age preference towards those above 50 years.^1^ The clinical presentation of primary breast chondrosarcomas is very similar to that of phyllodes tumours and metaplastic carcinomas. Histological examination remains an essential tool in differentiating between the three diseases. The absence of any epithelial component, and presence of chondroid areas with cellular atypia and pleomorphism in the breast specimen itself is the key factor in diagnosing such a disease.^5^

All the reported cases were treated with a radical surgical approach that entailed removal of all breast tissue. The role of axillary evacuation remains uncertain as all these cases had axillary dissection, nodal sampling or even sentinels node biopsy, which proved to be negative for malignancy.^6^ We therefore felt that axillary evacuation could be avoided with a more conservative approach.

The role of adjuvant therapy is very limited as chondrosarcomas are well known for being resistant to chemotherapy and radiotherapy. Surgical resection remains the only curative treatment option^1–3^ and adjuvant therapy was therefore not a valid option. The patient agreed with our decision and has remained disease free for 15 months.

## Conclusions

Surgical resection of primary breast chondrosarcoma remains the only curative option. However, the extent of such resection remains uncertain. Conservative breast surgery in these rare tumours could be a successful approach, especially when no axillary evacuation or adjuvant therapy is required.
